# Porcelain

**Published:** 1903-05-15

**Authors:** O. N. Lanphear


### PORCELAIN.

BY O. N. LANPHEAR, D.D.S.

Read before the Southwestern Michigan Dental Society, April 8, 1903.

"In framing an artist, art hath thus decreed,
To make some good but others to succeed."—Shakespeare.

A dentist must be an artisan, artist and physician, all in one. He must acquire delicacy of touch and manipulative skill of the very highest order; his eye must be trained to a keen perception of form, color and harmony, and his hand to execute the thoughts of his brain. He must be alive to the fact that porcelain is one of the "warmest" subjects with which the profession has to deal.

The possibilities of it for securing artistic effects in color, harmonious shading and satisfactory results in strength, articulation and adaptation, are carrying it in to the foremost ranks of dental prosthesis. A perfect porcelain incisor is no fit companion for one that is partly broken, decayed or discolored; and, since no art can make the defective tooth perfect and have the patient retain it, there is no alternative but to give so much imperfection to the artificial one as shall take away the striking contrast which offends our esthetic sense. "The dentist with an eye for the beautiful and a hand for executing, can reproduce nature's shapes and shades, cusps, sulci and contours with porcelain."

In this work there is no royal road. Method and detail are the principal factors. The essentials for satisfactory work are—

*First.*—An efficient system for manipulating the porcelain.

*Second.*—A good, high-fusing body.

*Third.*—Cleanliness, caution and accuracy in every operation.

Good crown and bridge-work is both, practical and possible with the different small furnaces manufactured at present. For my use, the Turner furnace (made in Chicago) is adequate. It will fuse the best grades of porcelain just as perfectly as any electric furnace. It is inexpensive, and occupies very little space. It can be used in any town and in any practice. Any good high-fusing body that has strength, and is not liable to lose its color in fusing. I find the S. S. White's new standard shade porcelains answer all purposes. Good results can not be expected if impurities are allowed to creep in, and all moisture should be eliminated or the anticipated pleasure may turn to pain upon seeing the finished piece. If a post, band and cap are fitted accurately to a root, and a veneer of the proper shade has been selected and soldered to the same, then the body can be packed to the proper form, carved to shape and fused.

With this form of crown, we can look for more satisfactory results than can be expected from the Logan Crown as ordinarily used. Where the remuneration is sufficient, good results may be obtained from the staining and changing of plate teeth. An over-lapping lateral or a worn cuspid can be made easily and does away with much of the artificial appearance of full dentures.

Fewer hours in the chair, more artistic work and less pain is the cry of the public. Fewer hours at the chair, more artistic work and more "payin" is the cry of the dentist. Then turn to porcelain, for it is the panacea.

DISCUSSION.

DR. J. M. THOMPSON, of Detroit: I like to talk of things near to my heart, but I would not have you think I have a porcelain heart. Porcelain work does not promise an easy time or short hours for the dentist; it is the most exacting work I ever undertook. Porcelain crowns adapted artistically and skillfully to certain cases are the acme of perfection, so far as cosmetic and useful results are concerned. They are more substantial and artistic than any Logan or other form of ready-made crown can be, and colors can be accurately matched. For inlays or porcelain fillings we are attaining a degree of perfection that makes this form of filling the standard of excellence.

Some workers mix the colors and get homogeneous effects, while others think they get more artistic color effects by laying in the different colors in strata as it were. A great variety of methods of securing the inlay matrix have been advocated, but I prefer the method of burnishing which I demonstrated in my clinic. The difficulties formerly met of securing a proper setting of the inlays with cement is now very well overcome by using a high grade cement and etching the surface of the inlay which comes in contact with the cement. And the principle of allowing the cement to crystallize under sufficient pressure to secure the best quality of hardness and adhesion.

Staining artificial teeth in crown, bridge and plate-work to match unusual conditions is very satisfactorily done now.

I have, I think, had very good success with porcelain inlays. I have lost but four inlays since I began using the material, and each of these cases can be accounted for because of the use of inferior cements.

The cement problem is the one most likely to bother the beginner in this work. I should advise any one undertaking this work to get a good cement and experiment with it until he knows just how to mix and use it to the best advantage for this purpose.

I enjoy doing porcelain work more than anything I do. To etch the inlay, cover the enameled surface with white wax and drop the inlay in hydrofluoric acid until it produces a rough surface.

Dr. S. M. Fowler: I use the Richmond porcelain face crown now altogether; it is artistic and stronger than any other crown made, in my judgment. I recently made a six-tooth porcelain bridge, which I think, is the best thing I have ever done. I am now using the Brewster body and a Pelton electric furnace and find that my work is definite and easy to what it used to be.

Dr. C. H. Warboys: I have had considerable experience with the low-fusing bodies for crown and inlay work, and I am not enthusiastic as to results. The inlays tend to spheroid and the colors run together so that there is no harmony with the color variations in the different parts of the tooth, and they do not refract the light as they should. I am now fusing Brewster's body in the small Downey gas furnace. I mixed some body with glycerine in place of water as an experiment and with this pasted attached two porcelain teeth together so tenaciously, that I could not separate them without using pliers.

I made an inlay some two years ago and set it, although I realized that it was inharmonious. I have since tried to get my patient to allow me to replace it with one of a better color, but he will not have it so. It is in a tooth from which the patient has bit out three gold fillings. I instructed him to bite this inlay out if he could, as I am anxious to make it over, but I presume I shall be compelled to wait some time, judging from the looks of the inlay the last time I saw it.

I have sometimes set a gold filling which has fallen out while finishing and find that a very excellent inlay can be made in this way.

Dr, Thompson: We should use great care in adjusting inlays with wedges as I have broken a beautiful inlay by wedging it too hard. An accident of this sort is very provoking.

Dr. Geo. Cook: I have in my mouth a lower molar tooth with a large distal cavity which extends into the occlusal surface. This tooth was filled with gold but was so sensitive to thermal changes, that I had it removed and a porcelain inlay inserted; this stopped the sensitiveness, but after breaking out three porcelain inlays, I concluded I would try something else. I had a gold inlay made and inserted and the tooth is not at all sensitive, and the inlay looks as as though it would last forever. This is a case where I think a gold inlay is superior to a porcelain inlay.

Dr. Taft: I most cordially endorse the porcelain idea now so generally discussed. For many purposes porcelain fillings are undoubtedly superior to gold. We should patiently go on experimenting until we shall get a material which shall meet our needs. Some will undoubtedly become more efficient than others, as with other forms of work. I predict that before long porcelain inlays will be commonly used. We should supply ourselves with the best materials and appliances available. With perfected appliance the time will come when we shall be able to produce satisfactory results in much less time than now. Just as we make gold crowns much more expeditiously now than formerly. We should keep up the standard of gold filling and develop the porcelain filling to its perfection.

